# Retraction: In vivo and in vitro effects of hyperplasia suppressor gene on the proliferation and apoptosis of lung adenocarcinoma A549 Cells

**DOI:** 10.1042/BSR-2018-0391_RET

**Published:** 2023-03-02

**Keywords:** A549 cells, Apoptosis, Gene silencing, Hyperplasia suppressor gene, Lung adenocarcinoma, Proliferation

This Retraction follows an Expression of Concern relating to this article previously published by Portland Press.

This article is being retracted from *Bioscience Reports* at the request of the Editor-in-Chief and the Editorial Board following receipt of a notification from a reader, alerting the Editorial Board to image duplications between Figures 1E and 1F in the *Bioscience Reports* article, and Figure 4B by Liu et al. 2019 (https://doi.org/10.1016/j.dld.2018.08.021).

The *Bioscience Reports* paper was also flagged in Labbé et al. 2019 (https://doi.org/10.1371/journal.pone.0213266) due to incorrectly listed sequence codes. The Editorial Office approached the authors for a response to this concern and did not receive a response.

The authors have been contacted with regards to the retraction, and have not responded to the Journal's queries and the concerns raised. Given the extent of the issues raised, the Editorial Board stand by the decision to retract the article.

